# 肿瘤患者新冠疫苗接种：临床研究进展与初步临床推荐

**DOI:** 10.3779/j.issn.1009-3419.2021.101.18

**Published:** 2021-06-20

**Authors:** 珞 王, 燕 徐, 路 张, 俊平 范, 瑞丽 潘, 京岚 王, 孟昭 王

**Affiliations:** 1 100730 北京，中国医学科学院，北京协和医学院，北京协和医院呼吸与危重症医学科 Department of Respiratory and Critical Care Medicine Hospital, Peking Union Medical College Hospital, Chinese Academy of Medical Sciences and Peking Union Medical College, Beijing 100730, China; 2 100730 北京，中国医学科学院，北京协和医学院，北京协和医院血液内科 Department of Hematopathology, Peking Union Medical College Hospital, Chinese Academy of Medical Sciences and Peking Union Medical College, Beijing 100730, China

**Keywords:** 新型冠状病毒肺炎, 疫苗, 肿瘤患者, Coronavirus disease 2019, Vaccines, Cancer patients

## Abstract

新型冠状病毒肺炎（coronavirus disease 2019，COVID-19，简称新冠肺炎）疫情对全球公众健康产生了严重影响。广泛接种新型冠状病毒疫苗（简称新冠疫苗）成为终止新冠疫情的最可能有效的手段之一。肿瘤患者在新冠肺炎疫情中感染风险大、严重事件发生率高、死亡率高，应是疫情防治的重点对象。目前肿瘤患者新冠疫苗接种研究证据尚缺乏。本文基于最新的临床研究，对肿瘤患者接种新冠疫苗的安全性和免疫效能相关数据进行初步综述。由于肿瘤患者的免疫功能情况复杂，本文对于应用不同肿瘤治疗方式及处于不同治疗阶段的患者提出初步的新冠疫苗接种建议。肿瘤患者新冠疫苗接种对新冠肺炎的预防极为重要，目前仍需大规模的临床研究和真实世界研究数据来制定更加精细的新冠疫苗接种方案。

## Abstract (en)

The pandemic of coronavirus disease 2019 (COVID-19) has had a serious impact on global health. COVID-19 vaccines may be one of the most effective measure to end the pandemic. High infection risk and higher serious incident and mortality rates have been shown in cancer patients with COVID-19. Therefore, cancer patients should be the priority group for COVID-19 prevention. Until now, data of COVID-19 vaccination for cancer patients is lacking. We review the interim data of safety and immune-efficacy of COVID-19 vaccination in cancer patients based on the latest studies. Due to the complicated immune systems of cancer patients caused by the malignancy and anticancer treatments, we proposed preliminary specific COVID-19 vaccination recommendations for cancer patients with different anticancer treatments and at different stages of the disease. Preventing COVID-19 with vaccinations for cancer patients is crucial, and we call for more large-scale clinical trials and real-world studies, for further COVID-19 vaccination recommendations development.

新型冠状病毒肺炎（coronavirus disease 2019，COVID-19，简称新冠肺炎），是由RNA病毒新型冠状病毒（severe acute respiratory syndrome coronavirus 2, SARS-CoV-2）引起的一种新发急性传染性疾病。新冠肺炎对全世界的公共卫生健康造成巨大影响，据世界卫生组织（World Health Organization, WHO）统计，截至2021年5月8日，共导致全球156, 496, 592人感染，其中3, 264, 143人死亡；截至2021年5月5日，全球共接种疫苗1, 171, 658, 745剂^[1]^。目前针对新冠肺炎仍缺乏确定有效的治疗手段，尚无经过严格“随机、双盲、安慰剂对照研究”证实的抗病毒药物可供使用^[2]^。通过新冠疫苗的大规模接种，建立全人群免疫屏障，可能是终止新冠疫情的最有效手段。

肿瘤是全球第二大死亡原因，人群中发病率和死亡率极高。根据世界卫生组织/国际癌症研究署（WHO/ International Agency for Research on Cancer, WHO/IARC）发布的《2020全球癌症报告》^[3]^，2018年全球新发癌症例数为1, 810万。由于肿瘤患者平均年龄大，并发症多，抗肿瘤治疗导致免疫功能抑制，因治疗需求反复出入医院，因此肿瘤患者在新冠肺炎疫情中感染风险大、严重事件发生率高、死亡率高^[4-7]^。因此，肿瘤患者的新冠肺炎预防极为重要，但是肿瘤患者新冠疫苗接种研究证据尚缺乏，相关研究正在开展。这部分人群的新冠疫苗接种策略需重点关注、精细制定。目前已有欧洲医学肿瘤学会^[8]^、癌症免疫治疗学会^[9]^、法国免疫治疗协会^[10]^和国家综合癌症网络^[11]^新冠疫苗接种咨询委员做出了关于肿瘤患者接种新冠疫苗的临床建议，为新冠疫苗的临床接种提供了一定的指导。

## 1 肿瘤合并新冠肺炎流行学特征

在新冠肺炎疫情初期，中国疾病预防控制中心对中国内地截至2020年2月11日确诊、疑似及临床诊断的72, 314例病例进行描述^[12]^，有107例（0.5%）基础疾病为癌症，其中6例发生死亡，粗死亡率为5.6%，高于整体人群粗病死率的2.3%。来自中国武汉的105例COVID-19癌症患者参与的多中心研究^[5]^结果显示，与没有癌症的COVID-19患者相比，癌症患者中观察到的死亡率更高（OR=2.34, 95%CI: 1.15-4.77, *P*=0.03），ICU入院率更高（OR=2.84, 95%CI: 1.59-5.08, *P* < 0.01），且至少有一种严重症状的发生率更高（OR=2.79, 95%CI: 1.74-4.41, *P* < 0.01）。来自全球的研究中也得到类似结果。COVID-19肿瘤联盟发起的登记研究中，纳入来自美国、加拿大和西班牙的928例肿瘤患者，242例（26%）患者为重症患者；132例（14%）患者需要ICU治疗，116例（12%）患者需要机械通气支持，121例（13%）患者死亡，且均在COVID-19诊断的30 d内^[6]^。英国的研究团队^[7]^针对活动性肿瘤的800例患者前瞻性研究中，226例（28%）患者死亡，其中211例（93%）患者死因归因于新冠肺炎。上述结果均提示：肿瘤患者在新冠肺炎疫情中症状重、严重事件发生率高、死亡率高，需重点关注。

## 2 新冠疫苗研发及应用现状

疫苗抗原和技术路线的选择是疫苗研发中的两个重点。新冠疫苗的抗原基础为SARS-CoV-2主要结构蛋白之一刺突蛋白（spike protein，S蛋白）。新冠疫苗的技术路线主要可分为病毒疫苗、病毒载体疫苗、基于核酸的疫苗、基于蛋白的疫苗四大类。根据WHO统计，截至2021年5月7日，处于临床试验阶段的候选新冠疫苗达97种，此外还有183种候选疫苗处于临床前评估阶段^[1]^。目前发表（包括正式发表和通过medRxiv预发表）Ⅲ期临床试验结果的新冠疫苗有5种，分别为病毒疫苗中的灭活病毒疫苗、基于核酸的疫苗中的mRNA疫苗，主要Ⅲ期临床试验结果见表 1。另外，WHO公开BBIBP-CorV（灭活疫苗，北京生物制品研究所/中国医药集团）的Ⅲ期临床数据：在18岁-59岁人群中，疫苗保护效力为78.1%^[1]^。根据美国食品药品监督管理局（Food and Drug Administration, FDA）数据^[13]^，Ad26.COV2.S疫苗预防中重度新冠肺炎的有效率约为66%。我国国家药品监督管理局（National Medical Products Administration, NMPA）附条件批准使用的新冠疫苗有5种，除了CoronaVac、BBIBP-CorV以外，NMPA也附条件批准SARS-CoV-2灭活疫苗（灭活疫苗，武汉生物制品研究所/中国医药集团）、Ad5载体COVID-19（腺病毒载体疫苗，康希诺生物股份公司/北京生物技术研究所）、ZF2001（重组蛋白疫苗，安徽智飞龙科马生物制药有限公司/中国科学院微生物研究所）注册申请。BBIBP-CorV、ChAdOx1 nCoV-19（也被称为AZD1222）、mRNA-1273、BNT162b2、Ad26.COV2.S（腺病毒载体疫苗，美国强生公司）获得WHO紧急授权批准使用^[1]^。

**表 1:** 新冠疫苗的保护力和安全性（截至2021年5月4日）已报道Ⅲ期临床试验结果的新冠疫苗 Efficacy and safety of COVID-19 vaccines: Data of reported phase 3 trials by May 4, 2021

研发路线	代表药物/研发团队	研究/发表阶段	实验组总人数（个）	保护效力（%）	严重病例（个）	严重不良反应^a^（个）	紧急授权
灭活病毒疫苗	CoronaVac/ 北京科兴生物制品有限公司^[14]^	Ⅲ/Ⅲ	4, 953	50.7	0	0	NMPA
腺病毒载体疫苗	Gam-COVID-Vac/ 俄罗斯加马利亚研究所^[15]^	Ⅲ/Ⅲ	16, 501	91.6	0	0	-
	ChAdOx1 nCoV-19/ 牛津大学/阿斯利康公司^[16]^	Ⅳ/Ⅲ	5, 807	70.4	0	1	WHO
mRNA疫苗	mRNA-1273/美国莫德纳公司/美国国家过敏和传染病研究所^[17]^	Ⅳ/Ⅲ	15, 210	94.1	0	0	WHO
	BNT162b2/德国BioNTech公司/辉瑞公司/中国复星医药^[18]^	Ⅳ/Ⅲ	21, 720	95	1	-	WHO
NMPA：国家药品监督管理局；WHO：世界卫生组织；^a^指证实与疫苗接种相关的严重不良反应病例个数；COVID-19：新型冠状病毒肺炎。							

## 3 肿瘤患者新冠疫苗接种研究现状

目前为止，肿瘤患者应用新冠疫苗的数据非常有限。现已公布临床试验结果的疫苗临床研究项目中，肿瘤患者以及接受免疫抑制、免疫调节治疗患者多不符合受试者纳入标准^[19]^，因此肿瘤患者入组困难，难以获得相关研究数据。相关临床试验中也较少针对肿瘤患者的疫苗接种有效性和安全性进行描述与分析。在BNT162b2新冠疫苗的研究中^[18]^，共纳入43, 540例受试者，其中3.7%的受试者有肿瘤病史，5例患者后期诊断COVID-19，其中1例为疫苗接种组，4例为对照组。多个肿瘤学术组织呼吁针对肿瘤患者开展新冠疫苗相关研究和评估，目前已有相关研究在开展，并有部分结果进行报告。

### 3.1 肿瘤患者新冠疫苗接种免疫效能评估

Monin等^[20]^报道了54名健康人群和151例肿瘤患者（实体瘤占63%，血液肿瘤占37%）应用BNT162b2新冠疫苗的免疫学评估结果，对于健康受试者，接种第1剂疫苗3周后，94%（32/34）检测到anti-S IgG抗体反应，接种过第2剂疫苗后，自接种后5周检测100%（12/12）产生抗体；实体瘤患者，接种第1剂疫苗3周后38%（21/56）产生抗体，接种过第2剂疫苗后，自接种后5周95%（18/19）产生抗体。血液系统肿瘤患者，接种第1剂疫苗3周后18%（8/44）产生抗体，接种过第2剂疫苗后，自接种后5周60%（3/5）产生抗体，提示血液肿瘤患者接种后免疫效能欠佳。Herishanu等^[21]^报道了慢性淋巴细胞白血病（chronic lymphoblastic leukemia, CLL）的167例患者接种了BNT162b2新冠疫苗的血清学反应，研究结果显示仅有39.5%（66/167）的患者检测到血清学反应，52例患者的反应率也显著低于配对的对照组（52% *vs* 100%）。接受治疗的患者整体的疫苗抗体反应率非常低[应用BTK抑制剂患者为16.0%；应用维奈托克（Venetoclax）±抗CD20单克隆抗体（抗CD20单抗）患者为13.6%]，尤其是12个月内接受抗CD20单抗的患者，无人对疫苗产生抗体反应。Bird等^[22]^评估93例多发骨髓瘤患者接种疫苗一剂21 d以后的血清学反应，结果显示52例（56%）患者检测到针对SARS-CoV-2 IgG抗体。注射BNT162b2疫苗组的IgG抗体阳性率为54%，ChAdOx1 nCoV-19疫苗组的IgG抗体阳性率为58%，两组无统计学差异（*P*=0.84）。疾病控制欠佳的患者（疗效评估为“稳定”或“进展”），IgG抗体阳性率为30%，低于疗效评估为“部分缓解”以上的人群（包括“完全缓解或非常好的部分缓解”IgG抗体阳性率为63%、“部分缓解”IgG抗体阳性率为75%）（*P*=0.004, 6）。正在接受针对骨髓瘤治疗的患者，IgG抗体阳性率低于未在接受治疗的患者（48% *vs* 74%, *P*=0.037）。Agha等^[23]^评估了67例血液系统肿瘤患者接受两剂mRNA疫苗后的血清学反应，结果显示53.5%（36/67）检测到针对SARS-CoV-2 IgG抗体，其中CLL患者的血清学反应率仅有23.1%（3/13），显著低于其他血液系统肿瘤患者的61.1%（33/54）（*P*=0.01）。

上述研究结果显示，实体瘤患者接种新冠疫苗后，其血清学反应弱于健康人群，实体瘤患者尽量接种两剂疫苗，方可实现一定程度的保护效能，而血液系统肿瘤患者接种新冠疫苗后，血清学反应更差，尤其是慢性淋巴细胞白血病的患者。抗肿瘤治疗对于新冠疫苗影响方面，目前相关研究提示，正在接受抗肿瘤治疗的患者较未接受抗肿瘤治疗的患者，血清学反应要弱，因此，尽可能地在治疗间期进行疫苗注射。不同的抗肿瘤治疗的差异方面，参考流感疫苗相关研究^[24]^，接受化疗的患者，接种疫苗后血清转化率和血清保护作用似乎比一般人群要低，而接受单药免疫检查点抑制剂（immune checkpoint inhibitor, ICI）治疗的患者则与正常人群相同。对于近1年内接受抗CD20单抗血液系统肿瘤的患者，由于药物作用导致的B细胞减少，将极大地影响疫苗介导的体液免疫效能^[21]^。

### 3.2 肿瘤患者新冠疫苗接种短期安全性评估

由于新冠疫苗应用时间尚短，且缺乏足够的肿瘤患者接种新冠疫苗后的安全性数据。在Monin等^[20]^的研究中，46%的肿瘤患者和62%的健康受试者在第1次接种后报道了局部和（或）全身副反应，29%的肿瘤患者和69%的健康受试者在第2次接种后报道局部和（或）全身副反应。Herishanu等^[21]^研究中，该组CLL人群中52例（31.1%）和56例（33.5%）在第1剂和第2剂接种后报道了局部反应，21例（12.5%）和39例（23.4%）报道了全身性反应。上述结果显示，肿瘤患者接种新冠疫苗的整体短期安全性与健康人群类似。肿瘤患者接受新冠疫苗后的长期安全性以及疫苗对于肿瘤治疗的影响，数据仍在积累中。既往肿瘤患者接种其他病毒疫苗的数据^[25]^也可以给我们一些提示，目前研究较多的是流感疫苗。根据肿瘤患者接种流感疫苗的数据^[26]^，除减毒活疫苗和具有复制能力的载体疫苗以外的病毒疫苗在常规手术、放化疗的肿瘤患者中是安全的。

免疫治疗，尤其是ICI是近年来新兴的抗肿瘤治疗策略，由于疫苗接种对人体的主要影响在于特异性体液免疫、细胞免疫的激活，因此疫苗安全性在接受ICI治疗的患者中尤为受关注。一项以色列的临床研究^[27]^纳入了2021年1月11日-2021年2月25日的170例肿瘤患者，他们均接受了ICI治疗，并在该项试验中进行BNT162b2疫苗接种。33例（19%）因担心疫苗的副作用拒绝接种。137例（81%）患者接受了第1剂疫苗接种，其中134例（98%）接受了第2剂疫苗接种。首剂后有3例患者死亡，其中1例归因于新冠肺炎，另2例因肿瘤进展而死亡。首次给药后最常见的副作用是局部反应，在134例患者中有28例（21%）报告了注射部位的疼痛。系统性副作用包括疲劳（5例，4%）、头痛（3例，2%）、肌肉疼痛（3例，2%）和发冷（1例，1%）。第2剂量疫苗后比第1剂量后观察到更多的全身和局部副作用。所报告的副作用均无需特殊干预措施。该临床试验中未观察到新的免疫相关副作用或现有免疫相关副作用的恶化。目前已有临床研究^[28, 29]^评估流感疫苗接种在接受ICI治疗的肿瘤患者的安全性以及对于免疫相关不良反应发生率的影响，研究结果显示此类患者应用流感疫苗无明显新的安全性事件，且没有增加免疫相关不良反应。综上，参照已有的临床证据和既往流感疫苗的相关研究结果，肿瘤患者接种新冠疫苗整体短期安全性与健康人群类似，未增加免疫相关不良反应，但长期安全性需要进一步的评估和总结。

### 3.3 肿瘤患者接种新冠疫苗意愿

一项来自法国4个癌症中心的横断面研究^[30]^，2020年11月11日-2020年12月12日期间共发放了1, 244例肿瘤患者的问卷调查，999例患者返回问卷，结果显示536例（53.7%）患者有意愿进行新冠疫苗接种，297例（29.7%）患者尚未做好接种准备，但是可能会改变想法，而166例（16.6%）患者无接种意愿。来自美国的另一项研究^[31]^，针对肿瘤患者及其照顾者进行问卷调查，并进行网络培训，在网络培训前接受问卷调查的205例参加者中，71%调研人群愿意接种新冠疫苗，24%不确定，而5%无接种意愿；接受网络培训后进行问卷调查的138例参加者中，82.5%调研人群愿意接种新冠疫苗，15.4%不确定，而2%无接种意愿，充分说明了针对新冠疫苗的患者教育，可以有效增加肿瘤患者及照顾者的新冠疫苗接种意愿。上述研究显示目前肿瘤患者半数以上有接种新冠疫苗的意愿，可通过有效的患者教育，提高此类患者的疫苗接种意愿。

## 4 肿瘤患者接种新冠疫苗的推荐

肿瘤患者在新冠肺炎疫情中易于感染，感染后严重事件发生率高、死亡率高。现有的新冠疫苗在肿瘤患者中应用的数据显示，肿瘤患者接种新冠疫苗后有不同程度的免疫应答，而且未观察到疫苗接种相关不良反应发生率增加。因此，建议肿瘤患者接种新冠疫苗。我国国家卫生健康委员会2021年3月29日颁布了《新冠病毒疫苗接种技术指南（第一版）》，技术指南中提及，目前尚无新冠病毒疫苗对于免疫功能受损人群（例如恶性肿瘤等）的安全性和有效性数据。该类人群疫苗接种后的免疫反应及保护效果可能会降低。对于灭活疫苗和重组亚单位疫苗，根据既往同类型疫苗的安全性特点，建议接种；对于腺病毒载体疫苗，既往无同类型疫苗使用的安全性数据，建议经充分告知，个人权衡获益大于风险后接种。欧洲医学肿瘤学会^[8]^、癌症免疫治疗学会^[9]^、法国免疫治疗协会^[10]^和国家综合癌症网络^[11]^等学术组织也做出了关于肿瘤患者接种新冠疫苗的临床建议。本文综合现有的临床研究及相关指南，给予初步的临床接种建议（表 2）。

**表 2:** 不同治疗方式与阶段肿瘤患者接种新冠疫苗的推荐 COVID-19 vaccination recommendations for cancer patients with different anticancer treatments and at different stages of the disease

肿瘤类型	治疗方式与阶段	接种时机	疫苗种类
实体肿瘤	手术	手术前1周以上或手术并发症恢复后	1.经WHO及所在国药品监督管理机构授权批准的疫苗； 2.避免活病毒疫苗； 3.谨慎应用腺病毒载体疫苗。
	放疗	任何时期	
	化疗	化疗前2周以上，化疗结束后1周-2周	
	靶向治疗（如EGFR-TKI）	任何时期	
	内分泌治疗	任何时期	
	免疫治疗（如ICI）	任何时期	
血液系统肿瘤	骨髓移植（自体、异体）	移植后3个月以上	
	细胞免疫治疗（如CAR-T）	治疗后3个月以上	
	化疗	化疗间期，中性粒细胞水平恢复后	
	靶向治疗（如抗CD20单抗）	抗CD20单抗结束治疗后6个月以上	
	表观遗传学治疗	任何时期	
筛查评估阶段		任何时期	
其他： 1.建议肿瘤患者的照料者和家庭成员及医护人员接种新冠疫苗； 2.肿瘤患者、照料者和家庭成员以及医护人员接种疫苗后仍需采取积极有效的公共卫生防控措施来预防新冠肺炎。			
EGFR-TKI：表皮生长因子受体酪氨酸激酶抑制剂；ICI：免疫检查点抑制剂；CAR-T：嵌合抗原受体T细胞。			

### 4.1 疫苗种类选择

首先，应选择WHO及所在国药品监督管理机构授权批准的疫苗；第二，避免活病毒疫苗。尽管WHO紧急授权和我国NMPA附条件批准的新冠疫苗研发路线并无减毒活病毒疫苗，但仍有活病毒疫苗项目进入临床试验阶段，肿瘤患者应避免接种；第三，谨慎应用腺病毒载体疫苗。自新冠疫苗在各国大规模接种，疫苗接种后产生严重不良反应的病例陆续被报道。其中多篇报道^[32-34]^提示腺病毒载体疫苗ChAdOx1 nCoV-19、Ad26.COV2.S可能与罕见的接种后血栓病例（包括肺栓塞、颅内静脉血栓、弥散性血管内凝血等）相关。相关研究提示，血栓形成机制为患者体内产生了血小板因子4-肝素复合物抗体（PF4-heparin抗体），该抗体介导PF4依赖性血小板活化。但是腺病毒载体疫苗接种如何导致PF4抗体产生尚不明确。由于肿瘤细胞产生的炎症因子、促凝物质，以及它与其他细胞的相互作用，使肿瘤患者处于高凝状态，因此，在ChAdOx1 nCoV-19、Ad26.COV2.S疫苗接种后血栓产生机制完全明确前，建议肿瘤患者谨慎接种该研发路线的新冠疫苗。

### 4.2 新冠疫苗接种时机

针对不同治疗方式和阶段的肿瘤患者，我们推荐不同的新冠疫苗接种时机。

#### 4.2.1 实体瘤患者

① 手术患者：对于尚未接受手术的患者，我们建议接种新冠疫苗1周后行手术治疗，而已接受手术的患者，应等待术后恢复、手术并发症缓解后再进行疫苗接种。由于肿瘤患者手术为限期手术，应充分评估所处地区新冠疫情及所患肿瘤的生物学行为后，决定新冠疫苗接种时机；②放疗患者：局部放疗和病毒疫苗接种两者不会造成相互影响，因此采取单独放疗治疗肿瘤的患者可在治疗任何时期接受疫苗接种，而因为疫苗接种是当前预防新冠病毒感染的最重要手段，所以建议尽早接种；③化疗患者：患者接受化疗后会出现明显的骨髓抑制、免疫功能受损。因此疫苗接种后，机体无法正常免疫应答，无法产生高滴度的保护性抗体。我们建议新冠疫苗接种应至少在化疗前2周或化疗结束后2周进行。若仍不可行，根据有限的流感疫苗接种数据^[35, 36]^，可选择在化疗结束后1周-2周时接种；④靶向治疗、免疫治疗、内分泌治疗的任何阶段均可接受新冠疫苗接种，同样，我们建议尽早接种来避免感染新冠肺炎。

#### 4.2.2 血液系统肿瘤患者

① 骨髓移植及细胞免疫治疗患者：前者包括异体及自体骨髓干细胞移植。后者如嵌合抗原受体T细胞（chimeric antigen receptor T cell, CAR-T）免疫疗法等。该类患者在骨髓移植及细胞免疫治疗时会出现严重的骨髓抑制。因此应当在全身免疫系统重建后，再进行新冠疫苗接种。重建时间一般为3个月；②化疗：血液系统肿瘤患者接受的化疗方案一般强于实体肿瘤患者，化疗后较多出现IV度骨髓抑制，且化疗总疗程更长。在化疗的实体瘤患者新冠疫苗接种推荐基础上，建议血液系统肿瘤患者化疗间期，外周血中性粒细胞水平恢复时，进行疫苗接种；③靶向治疗：目前，以抗CD20单抗为主要代表的靶向治疗，在血液系统肿瘤中占有重要地位。根据前文所述，应用抗CD20单抗治疗的血液系统肿瘤患者新冠疫苗接种免疫效能评估，有限的研究数据表明：由于抗CD20单抗治疗后B细胞显著减少，疫苗介导的体液免疫受损，机体几乎无法产生保护性抗体。因此，建议结束抗CD20单抗6个月后可接种新冠疫苗。而对于需要长期抗CD20单抗治疗，持续保持极低B细胞水平以维持疾病缓解的患者，我们不建议接种新冠疫苗；④表观遗传学治疗的任何阶段均可接受新冠疫苗接种。

#### 4.2.3 其他

① 建议肿瘤患者的照料者和家庭成员及医护人员接种新冠疫苗；②尽管肿瘤患者可接种新冠疫苗，仍建议肿瘤患者、照料者和家庭成员以及医护人员采取积极有效的公共卫生防控措施来预防新冠肺炎。

肿瘤患者的新冠肺炎防治是重要公共卫生问题。接种新冠疫苗、建立全人群免疫屏障是新冠疫情防控的核心措施。目前新冠疫苗在肿瘤患者中的有效性、安全性数据较为缺乏。根据目前有限的证据，推荐肿瘤患者在合适的时机接种新冠疫苗。目前迫切需要大规模的临床研究并积攒有效的真实世界研究数据，为肿瘤患者新冠疫苗接种提供更多的安全性和有效性证据，以制定更加精细的新冠疫苗接种指导方案。
